# Phosphorylation of SNAP-25 at Ser187 is enhanced following its cleavage by Botulinum Neurotoxin Serotype A, promoting the dominant-negative effect of the resulting fragment

**DOI:** 10.1371/journal.ppat.1013604

**Published:** 2025-10-14

**Authors:** Dilara Koc, Sena Ezgin, Ebru Kavakli, Krishna P. Kota, Edanur Sen, Christopher Mahone, Mary Ellen Palko, Lisa H. Cazares, Tolga Can, Lino Tessarollo, Sina Bavari, Antoine Marion, Erkan Kiris

**Affiliations:** 1 Department of Biological Sciences, Middle East Technical University, Cankaya, Ankara, Turkey; 2 Countermeasures Division, United States of America Army Medical Research Institute of Infectious Diseases, Frederick, Maryland, United States of America; 3 Neural Development Section, Mouse Cancer Genetics Program, Center for Cancer Research, NCI, Frederick, Maryland, United States of America; 4 Department of Computer Engineering, Middle East Technical University, Ankara, Turkey; 5 Department of Chemistry, Middle East Technical University, Cankaya, Ankara, Turkey; 6 Meddenovo Drug Design, Villeurbanne, France; Boston Children's Hospital, UNITED STATES OF AMERICA

## Abstract

Botulinum Neurotoxin Serotype A (BoNT/A), responsible for most human botulism cases, inhibits neurotransmitter release by cleaving the target protein SNAP-25. Previous literature demonstrated that BoNT/A mediated cleavage of a small subset of the SNAP-25 pool, resulting in SNAP-25 (1–197) fragments, is sufficient to block exocytosis. SNAP-25 (1–197) potentially competes against intact SNAP-25 for SNARE complexes and blocks neurotransmission through a dominant-negative mechanism. However, how a tiny fraction of cleaved SNAP-25 efficiently outcompetes a large pool of intact SNAP-25 remains unknown. Here, we examined the importance of SNAP-25 phosphorylation at Ser187 residue, located in the C-terminus SNARE domain, in the context of BoNT action. Our results demonstrated that Ser187-phosphorylated SNAP-25 can be efficiently cleaved in cells. Importantly, BoNT/A-cleaved SNAP-25 fragments in neuronal and non-neuronal cells are heavily phosphorylated at Ser187 and localized on the cell membrane. SNAP-25 (1–197) binds to syntaxin-1A, and the interaction is enhanced by Ser187 phosphorylation. We also found that SNAP-25 (1–197) survives longer than the BoNT/A enzymatic component itself in cells. Molecular modeling suggested that SNAP-25 (1–197), phosphorylated or not, forms stable SNARE complexes; however, Ser187 phosphorylation induces local changes in surface electrostatic potential and dynamics of the complex. This study characterizes the molecular mechanism underlying the dominant-negative effect of SNAP-25 (1–197) on neurotransmission. This research could have implications for the future development of BoNT/A inhibitors and the generation of new BoNT/A clinical formulations by regulating the abundance of Ser187 phosphorylation in cleaved SNAP-25 fragments.

## Author summary

Botulinum Neurotoxins (BoNT) are bacterial toxins causing botulism disease. BoNT/A mediated cleavage of a minority of the SNAP-25 is sufficient to block neurotransmission. Our work here uncovers the molecular mechanism underlying the dominant negative effect of BoNT/A cleaved SNAP-25, SNAP-25 (1–197), on neuroexocytosis. We report that SNAP-25 (1–197) fragments are efficiently phosphorylated at the Ser187 residue in cells. SNAP-25 (1–197) has a long half-life in the cytosol, during which efficient Ser187 phosphorylation can contribute to exocytosis inhibition by providing a competitive advantage over WT SNAP-25 for SNARE complex formation. This study highlights Ser187 phosphorylation of SNAP-25 (1–197) as a potential therapeutic target for modulating the biological effects of BoNT/A, offering implications for biodefense and the development of novel BoNT/A formulations.

## Introduction

Botulinum neurotoxins (BoNTs) are highly toxic and potentially lethal molecules that cause botulism disease through selective mechanisms of action [1]. BoNTs block exocytosis at neuromuscular junctions, and there are no approved therapeutics to neutralize them once they are settled in neurons [2]. When administered in localized, small doses, these toxins are highly useful therapeutics with expanding cosmetic and clinical applications [3]. However, BoNTs are Category A bioterror agents, and there are concerns regarding accidents in clinics, highlighting the importance of better understanding the intoxication and recovery mechanisms [4]. Human botulism is caused by serotypes/A,/B,/E, and/F [5], and BoNT/A is responsible for most cases [6]. Serotypes/A and/E target the same protein, SNAP-25, in cells but with strikingly different outcomes [7]. The toxic effect of BoNT/E is limited to days, while BoNT/A effects could last months [8]. Such differences have been attributed to differences in the longevity of the enzymatic components (light chains; LC) of/A and/E [9]. However, it has long been suggested that SNAP-25 fragments generated by the toxin might also contribute to the observed toxicity differences through a dominant-negative mechanism as long as they persist in the cytoplasm [10–12].

Multiple groups have demonstrated that BoNT/A-mediated cleavage of only a tiny fraction of SNAP-25 is sufficient to cause significant inhibition of neuroexocytosis [10,13–17]. For example, BoNT/A-mediated truncation of less than 3% of total SNAP-25 was observed to inhibit synaptic activity in neurons by over 70% [18]. On the contrary, BoNT/E-mediated cleavage of SNAP-25 levels correlates well with exocytosis inhibition [13,14]. SNAP-25 cleaved by BoNT/A (SNAP-25 (1–197)), but not that of BoNT/E (SNAP-25 (1–180)), has been proposed to function as a dominant-negative to inhibit neurotransmission [10–12]. Several lines of evidence support this notion. First, functional SNARE complex formation and exocytosis require intact SNAP-25 [19,20], and unlike BoNT/E-truncated SNAP-25, BoNT/A-truncated SNAP-25 can still form stable SNARE complexes [16,21]. Second, the presence of SNAP-25 (1–197) alone, independently from the presence of the toxin, is sufficient to inhibit exocytosis [22–24]; however, SNAP-25 (1–180) does not inhibit exocytosis [23]. Third, co-administration of BoNT/A and E leads to short-term paralysis, a typical effect of BoNT/E in humans [10,25], although data in rodent models provided conflicting results [26]. Fourth, SNAP-25 (1–197) has been observed to stay on plasma membranes, positioned appropriately for SNARE complex formation, but inactivates complexes, inhibiting neurotransmitter release [13,27–29]. Taken together, SNAP-25 (1–197) may compete with intact SNAP-25 for SNARE complexes and thereby inhibit exocytosis [12–14]. However, how small amounts of cleaved SNAP-25 efficiently outcompete a large pool of intact SNAP-25 to extensively inhibit synaptic release remains unknown.

Phosphorylation is a crucial post-translational mechanism that tightly regulates neuronal SNARE protein functions [30,31]. SNAP-25 phosphorylation at Serine187, the only reported phosphorylation site in the SNARE domain at the C-terminus, has particularly important functional roles [32]. Studies utilizing a genetically modified mouse model in which the SNAP-25 Ser187 residue is replaced with alanine suggested that the phosphorylation has functional roles *in vivo*, as demonstrated by defects in serotonin and dopamine release [33], working memory [34], and basal synaptic transmission [35], as well as epilepsy-related seizures [36]. *In vitro* studies utilizing various cell lines suggested that the Ser187 phosphorylation of SNAP-25 increases secretion [32,37–40]. However, these findings have been challenged, suggesting that Ser187 phosphorylation is not crucial for releasing neurotransmitters [41,42]. While its role in exocytosis is unclear, multiple groups have established that the Ser187 phosphorylation enhances syntaxin/SNAP-25 interaction, leading to enhanced SNARE complex assembly [38,40,43]. The SNARE domain of SNAP-25 in the C-terminus is crucial for BoNT/A binding, and BoNT-mediated truncation of 9 amino acids from this terminal is sufficient to inhibit neurotransmitter release. However, there are no direct reports evaluating the importance of Ser187 phosphorylation of WT SNAP-25 for toxin-mediated cleavage and its potential functional roles in the cleaved fragment.

In this study, we utilized various immortalized cell lines as well as mouse and human embryonic stem cell (ESC)-derived motor neurons and determined that Ser187-phosphorylated SNAP-25 protein in cells is efficiently cleaved by BoNT/A. Importantly, our work demonstrated that BoNT/A-cleaved SNAP-25 fragments in both neuronal and non-neuronal cells are heavily phosphorylated at Ser187 compared to wild-type (WT) SNAP-25. SNAP-25 (1–197) appears to be a better substrate for Ser187 phosphorylation, and we did not observe a similar effect on the other main SNAP-25 phosphorylation residue, Thr138 [38,40,44], suggesting the observed effect is Ser187-specific. Membrane fraction analyses revealed membrane localization of both WT SNAP-25 and SNAP-25 (1–197); however, membrane-localized SNAP-25 (1–197) exhibits far greater Ser187 phosphorylation. We also detected that cellular SNAP-25 (1–197) persists longer than BoNT/A LC. SNAP-25 (1–197) binds to syntaxin-1A, and the interaction is enhanced by Ser187 phosphorylation. Molecular modeling identified that SNAP-25 (1–197), phosphorylated or not, still engages in forming stable SNARE complexes with no significant change. Phosphorylation at Ser187 in SNAP-25 (1–197), however, induces local changes in the electrostatic signature, rigidity, and dynamics of the complex, which likely play a role in the dominant-negative mechanism of exocytosis. This study is the first significant examination of the importance of SNAP-25 phosphorylation regarding BoNT/A action, and the results suggest Ser187 phosphorylation of SNAP-25 as a potential therapeutic target to modulate the biological effects of BoNT/A.

## Results

### BoNT/A holotoxin-cleaved SNAP-25 exhibits higher phosphorylation levels of Ser187

**Fig 1:**
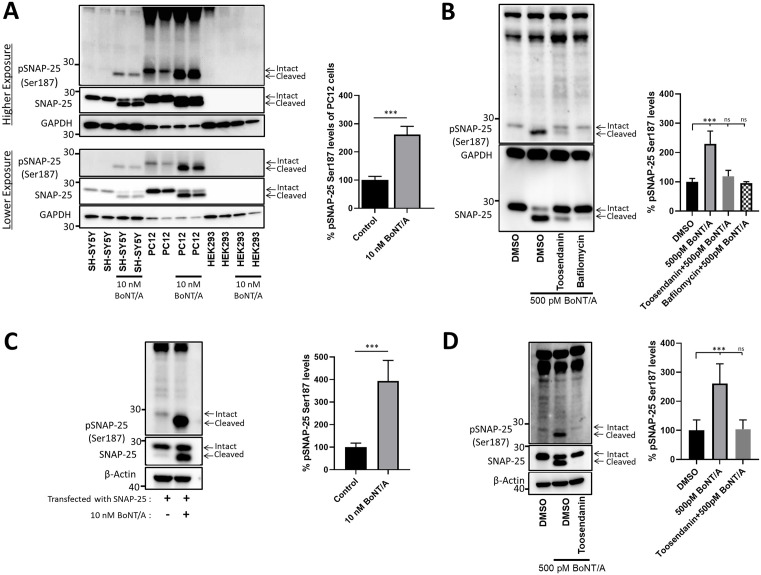
BoNT/A-cleaved SNAP-25 is heavily phosphorylated at the Ser187 residue. **(A)** SH-SY5Y cells, PC-12 cells, and HEK293 cells were treated with 10 nM BoNT/A holotoxin for 96 hours. Immunoblots, shown in lower and higher exposures, include two technical replicates for each condition and are representative of 3 independent biological replicates. The bar graph demonstrates the % of total pSNAP-25 Ser187 phosphorylation levels of PC-12 cells, normalized to total SNAP-25 levels. **(B)** mESC-derived motor neurons were treated with 500 pM BoNT/A for 4 hours, and Toosendanin and Bafilomycin were utilized as BoNT inhibitor controls. Representative blots of 3 independent experiments are shown. **(C)** HEK293 cells were transfected with WT SNAP-25 plasmid for 4 hours, followed by 10 nM BoNT/A holotoxin treatment for 72 hours. Western blot images are representative of 4 independent biological replicates. **(D)** hESC-derived motor neurons were treated with 500 pM BoNT/A holotoxin for 4 hours, and Toosendanin was utilized as a BoNT/A inhibitor. The immunoblots are representative of 5 independent biological replicates. GAPDH and β-Actin served as loading controls. In all conditions, the extent of % pSNAP-25 Ser187 phosphorylation levels was measured, and normalized to total SNAP-25 levels. *: P ≤ 0.05; **: P ≤ 0.01; ***: P ≤ 0.001.

We sought to determine SNAP-25 phosphorylation on Ser187 in cultured PC12, SH-SY5Y, and HEK293 cells before and after BoNT/A holotoxin intoxications. Additionally, we assayed mESC and hESC-derived motor neurons, as BoNT/A naturally targets motor neurons [45]. Our data shows that SNAP-25 (1–197) exhibits higher Ser187 phosphorylation levels in SH-SY5Y cells compared to uncleaved (intact; WT) SNAP-25 (Fig 1A). We quantified pSNAP-25 Ser187 levels in PC12 cells, and SNAP-25 (1–197) exhibits statistically significantly higher Ser187 phosphorylation (Fig 1A). HEK293 cells did not exhibit detectable endogenous SNAP-25 and served as a control within the same experimental set-up (Fig 1A). SNAP-25 (1–197) in mESC-derived motor neurons also exhibited statistically significantly higher Ser187 phosphorylation as compared to non-treated condition (Fig 1B). Both Toosendanin and Bafilomycin are well-established BoNT/A-mediated SNAP-25 cleavage inhibitors and served as controls. Further, we transiently transfected SNAP-25 in HEK293 cells and intoxicated the cells with BoNT/A holotoxin, and the results demonstrate that SNAP-25 (1–197) generated by BoNT/A cleavage is heavily phosphorylated at Ser187 residue (Fig 1C). To explore this phenomenon in physiologically relevant human neurons, we generated hESC-derived motor neurons, and our data demonstrated statistically significantly increased Ser187 phosphorylation of SNAP-25 (1–197) (Fig 1D). Overall, we demonstrated that SNAP-25 (1–197) fragments are heavily phosphorylated at Ser187 residue, compared to WT SNAP-25, in multiple cell-based analyses.

### Phosphorylation at the Ser187 residue does not inhibit SNAP-25 cleavage by BoNT/A in cells

**Fig 2:**
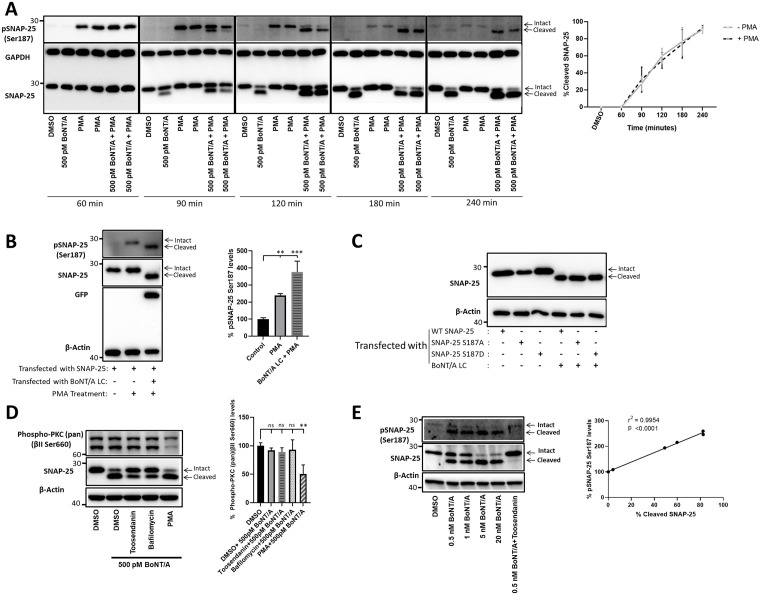
Ser187 phosphorylated SNAP-25 is efficiently cleaved in cells. **(A)** mESC-derived motor neurons were treated with 1 µM PMA for 30 min and then intoxicated with 500 pM BoNT/A holotoxin. Cells were collected at the indicated time points, respectively to the intoxication start time point, processed, and subjected to western blotting. Representative blots include two technical replicates for each condition and are representative of 3 independent biological replicates. The graph represents quantified % SNAP-25 cleavage in the presence and absence of PMA treatment at the indicated time points **(B)** HEK293 cells were transfected with WT SNAP-25 and/or YFP-tagged BoNT/A LC plasmids for 18 hours (n = 3). 1 µM PMA was supplied to PMA+ samples 1 hour following the transfection initiated. **(C)** WT SNAP-25, SNAP-25 S187A, and SNAP-25 S187D plasmids were individually or co-transfected with BoNT/A LC in HEK293 cells for 18 hours (n = 3). **(D)** mESC-derived motor neurons were treated with 500 pM BoNT/A holotoxin for 4 hours (n = 3). Toosendanin and Bafilomycin, well-known BoNT inhibitors, and PKC activator PMA were used as controls and added to the culture 30 min before intoxication started. **(E)** mESC-derived motor neurons were treated with increasing concentrations of BoNT/A holotoxin (0.5 nM – 20 nM) for 4 hours, and Toosendanin was supplied to the relevant sample 30 min before the intoxication (n = 3). At the end of each experiment, cell lysates were prepared and subjected to immunoblotting. ns: non-significant; *: P ≤ 0.05; **: P ≤ 0.01; ***: P ≤ 0.001.

We next sought to determine whether Ser187-phosphorylated SNAP-25 protein gets cleaved less efficiently in cells. To do so, we have utilized a PMA chase experiment to induce Ser187 phosphorylation in mESC-derived motor neurons and collected cells at various time points. PMA is a well-known activator of the PKC pathway and has been shown to induce Ser187 phosphorylation of SNAP-25 [46,47]. We treated the cells with PMA for 30 minutes before intoxication and then intoxicated with 500 pM BoNT/A holotoxin. 60 minutes after intoxication, there was no BoNT/A-mediated cleavage, but a robust increase in Ser187 phosphorylation was evident, suggesting that Ser187 phosphorylation was achieved before the toxin’s proteolytic activity began. With increased time points, it was clear that Ser187-phosphorylated SNAP-25 can be efficiently cleaved (120 min time point) (Fig 2A). Interestingly, with further time points, consistent with Fig 1, cleaved SNAP-25 exhibits higher Ser187 phosphorylation. We quantified % SNAP-25 cleavage in the presence and absence of PMA treatment at the indicated time course experiments, utilizing the data from 3 independent experiments, and the results indicate that there is no statistically significant difference between conditions with/without PMA at specific time points (Fig 2A, graph). We then tested these observations in HEK293 cells transfected with plasmids encoding SNAP-25 and BoNT/A LC. PMA treatment led to a significant increase in Ser187 phosphorylation, and BoNT/A LC transfection in PMA-treated conditions resulted in the cleavage of all the SNAP-25 (Fig 2B). Similar to Fig 2A, BoNT/A LC-cleaved SNAP-25 also exhibited significantly higher Ser187 phosphorylation levels. Additionally, we transfected plasmids encoding BoNT/A LC, WT SNAP-25, Ser187A (non-phosphorylatable), and Ser187D (a phosphomimetic variant) SNAP-25 in HEK293 cells and found that regardless of Ser187 phosphorylation or modification status, BoNT/A-mediated cleavage is not affected (Fig 2C). Collectively, the data suggest that Ser187-phosphorylated SNAP-25 can be efficiently cleaved in cells.

### Increased SNAP-25 phosphorylation in BoNT/A-treated cells is not due to the activation of PKC by BoNT/A holotoxin

Previous literature demonstrated that Ser187 phosphorylation of SNAP-25 is mediated by the kinase PKC [32], and therefore, we next asked whether PKC is activated due to BoNT/A holotoxin exposure. Among the PKC isoforms, nPKCε has been identified as the isoform mediating Ser187 phosphorylation of SNAP-25 [48]. To detect PKC activity changes, we utilized phospho-PKC (pan) (βII Ser660) antibody (Cell Signaling Technology, 9371, RRID: AB_2168219), which detects a number of PKC isoforms, including the epsilon isoform, a critical phosphorylation event for PKC activity [49]. We intoxicated mESC-derived motor neurons with 500 pM BoNT/A holotoxin under various control and experimental conditions, such as BoNT/A inhibitors Toosendanin and Bafilomycin, and the PKC activator PMA. We did not observe an apparent change in PKC (pan) (βII Ser660) phosphorylation in toxin-treated conditions, though PMA-mediated PKC activation [37,39] affected the PKC phosphorylation significantly (Fig 2D). Finally, we examined whether the observed Ser187 phosphorylation of SNAP-25 correlates with the BoNT/A concentration (Fig 2E). We evaluated the effects of increasing doses of BoNT exposure on the Ser187 phosphorylation of SNAP-25, and the results suggest that the proportions of pSNAP-25 Ser187 levels and cleaved SNAP-25 correlate (Fig 2E). These results suggest that BoNT/A holotoxin does not directly induce Ser187 phosphorylation of SNAP-25, suggesting that PKC is not activated due to the toxin exposure. It is important to note that BoNT/A LC transfection (as shown in Fig 2B) rather than holotoxin intoxication also leads to increased Ser187 phosphorylation in the cleaved fragment, indicating both holotoxin intoxication and LC transfection lead to the same result in terms of SNAP-25 Ser187 phosphorylation. Overall, this data suggests that BoNT/A-mediated removal of 9 amino acids may better expose the Ser187 residue for basal PKC activity-mediated phosphorylation.

### SNAP-25 (1–197) fragment is a better substrate for Ser187 phosphorylation

**Fig 3:**
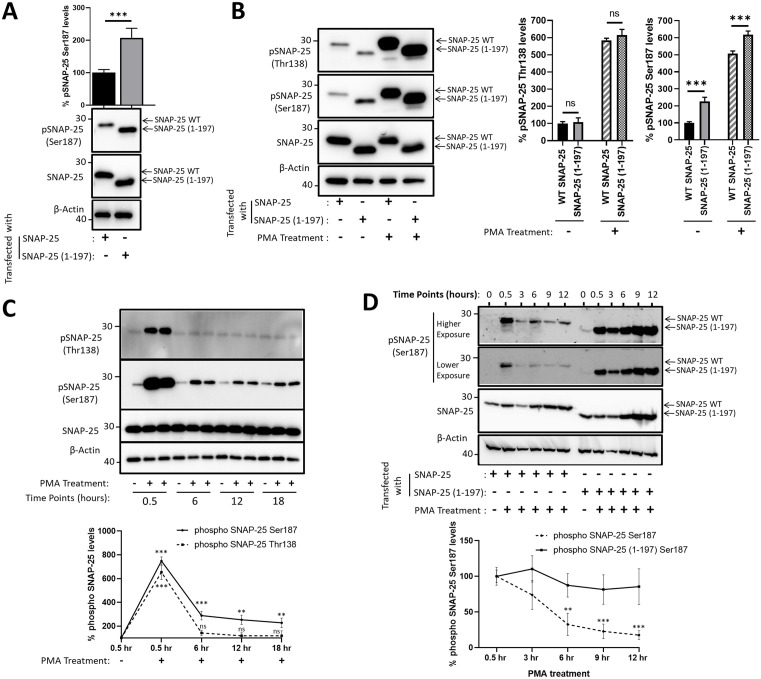
SNAP-25 (1-197) is a better substrate for Ser187 phosphorylation. HEK293 cells were transfected with either WT SNAP-25 or SNAP-25 (1-197) for 18h, and treated without **(A)** (n = 4), and with **(B)** (n = 4) PMA, to determine SNAP-25 Ser187 phosphorylation levels via western blotting. PMA was supplied to the cells 1 hour following the transfection initiated. Data was normalized to respective loading controls, WT SNAP-25 or SNAP-25 (1-197). **(C)** To measure the stability of Ser187 phosphorylation as compared to that of Thr138, PC12 cells were treated with 1 µM PMA, and samples were collected at the indicated time points (0.5h - 18h) following the PMA treatment, and were analyzed with immunoblotting (n = 3). **(D)** HEK293 cells were transfected with either WT SNAP-25 or SNAP-25 (1-197) for 18h and treated with 1 µM PMA. The samples were collected at indicated time points (0.5h – 12h) following the PMA treatment and subjected to western blotting (n = 3). ns: non-significant; *: P ≤ 0.05; **: P ≤ 0.01; ***: P ≤ 0.001.

Next, we explored whether SNAP-25 (1-197) gets more efficiently phosphorylated at Ser187 than WT SNAP-25 in cells. Our analyses involving HEK293 cells transfected with either WT SNAP-25 or SNAP-25 (1-197) suggest that SNAP-25 (1-197) is more efficiently phosphorylated at Ser187 compared to WT SNAP-25 (Fig 3A). In a similar experimental set-up, we explored the effects of PMA treatment on the phosphorylation levels of Ser187 and Thr138, i.e., the second phosphorylation residue of SNAP-25 with functional impact [38,40,44], in both WT SNAP-25 and SNAP-25 (1-197) in HEK293 cells. The data shows that PMA treatment leads to higher Ser187 and Thr138 phosphorylation, however, we have not detected a significant change in Thr138 phosphorylation levels between WT SNAP-25 and SNAP-25 (1-197) in PMA-treated and untreated conditions (Fig 3B). On the other hand, rates of Ser187 phosphorylation are statistically greater in SNAP-25 (1-197) compared to WT SNAP-25, suggesting that the increased phosphorylation is Ser187-specific (Fig 3B). Although PKA has been identified as the main kinase phosphorylating Thr138 [50], PKC has been reported to also phosphorylate Thr138, though Ser187 is the preferred PKC substrate [51]. Therefore, it is not surprising that PMA treatment led to elevated Thr138 phosphorylation of SNAP-25. Following, we explored the stability of Ser187 phosphorylation compared to Thr138 phosphorylation of endogenous SNAP-25 protein in PC12 cells, treated with or without PMA, at various time points (0.5, 6, 12, and 18 hours), and the data suggest that Ser187 phosphorylation persists longer than Thr138 phosphorylation (Fig 3C). Finally, we sought to compare Ser187 dephosphorylation level differences between WT SNAP-25 and SNAP-25 (1-197). To do so, we utilized HEK293 cells transfected with either WT SNAP-25 or SNAP-25 (1-197) for 18 hours, then treated with and without PMA, and collected the samples at various time points (up to 12 hours) (Fig 3D). Our results suggest that while WT SNAP-25 gets efficiently de-phosphorylated, Ser187 phosphorylation persists in SNAP-25 (1-197) with relatively less de-phosphorylation. Due to transient transfection, both WT SNAP-25 and SNAP-25 (1-197) protein levels increase during experimental duration; however, Ser187 phosphorylation in SNAP-25 (1-197) correlates with increasing SNAP-25 (1-197) levels (Fig 3D), consistent with an increased preference for phosphorylation of Ser187 in SNAP-25 (1-197).

### SNAP-25 (1–197) is localized to the membrane, and the fragments are heavily phosphorylated at Ser187

**Fig 4:**
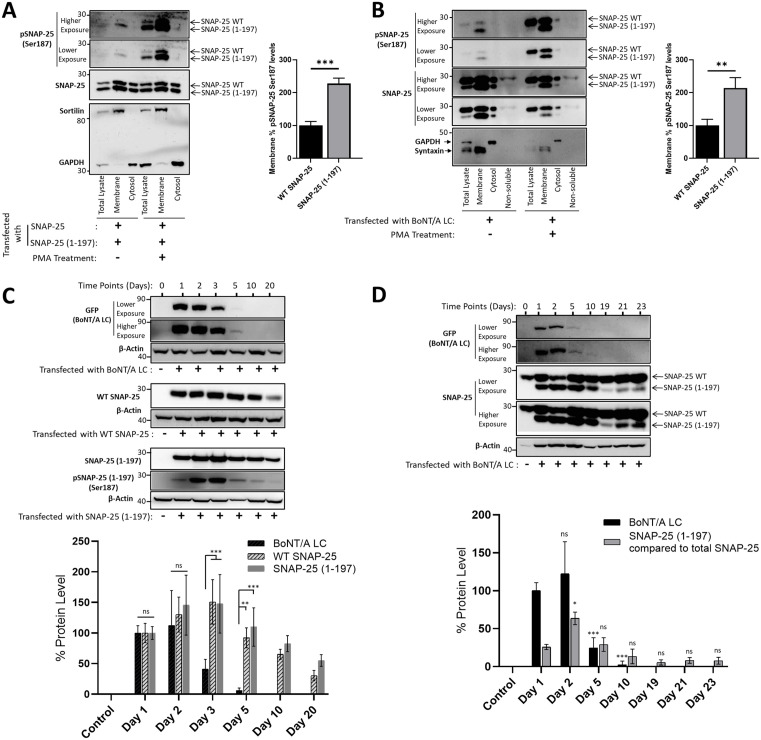
SNAP-25 (1-197) localizes to the membrane and is heavily phosphorylated at Ser187. **(A)** Subcellular distribution of SNAP25 and SNAP-25 (1-197) was analyzed by cell fractionation. HEK293 cells were co-transfected with WT SNAP-25 and SNAP-25 (1-197) and treated with and without 1 µM PMA for 1.5 hours. Cells were homogenized, followed by membrane extraction. The total lysate, as well as the soluble membrane and the cytosolic fractions, were analyzed by western blotting with the indicated antibodies. Data representative of 3 independent experiments. The bar graph demonstrates % pSNAP-25 Ser187 levels on the membrane upon PMA treatment, normalized to respective loading controls, WT SNAP-25 or SNAP-25 (1-197). **(B)** PC12 cells, which endogenously express SNAP-25, were transfected with BoNT/A LC in conditions to achieve a low-level SNAP-25 cleavage and treated with and without 1 µM PMA for 1.5 hours. The total lysate, the soluble membrane, cytosol, and non-soluble fractions were subjected to western blotting with the indicated antibodies. Representative blots of 3 independent experiments are shown. % pSNAP-25 Ser187 levels on the membrane upon PMA treatment were calculated upon normalization to respective loading controls, WT SNAP-25 or SNAP-25 (1-197), which is presented in the bar graph. **(C)** BoNT/A - cleaved SNAP-25 is detected in HEK293 cells longer than BoNT/A LC. HEK293 cells were transiently transfected with plasmids encoding YFP-tagged BoNT/A LC (n = 4), WT SNAP-25 (n = 3), or SNAP-25 (1-197) (n = 3), and cell lysates were collected at the indicated time points and analyzed with western blotting with the specified antibodies. Western blot band quantification results were normalized to loading controls (β-Actin) that were reprobed in each blot, and the results are presented as mean ± SD. **(D)** SNAP-25 (1-197) persists longer than BoNT/A LC in PC12 cells. PC12 cells were transiently transfected with YFP-tagged BoNT/A LC, samples were collected at the indicated time points, and subjected to immunoblotting. BoNT/A LC (detected by GFP) levels were determined upon normalization to the loading control. % SNAP-25 (1-197) compared to total SNAP-25 was calculated by measuring the intensities of SNAP-25 WT and SNAP-25 (1-197) bands in each time point. The statistical significance shown in the bar graphs represents the comparison of protein levels at each time point to the Day 1 level. ns: non-significant; *: P ≤ 0.05; **: P ≤ 0.01; ***: P ≤ 0.001.

We next set out to determine the subcellular distribution of SNAP-25 (1–197) and whether it differs from that of SNAP-25. SNAP-25 participates in the SNARE complex on the membrane [52], and therefore we have evaluated whether the BoNT/A-cleaved fragment stays on the membrane using membrane extraction analyses in both HEK293 (Fig 4A) and PC12 cells (Fig 4B). HEK293 cells, co-transfected with WT SNAP-25 and SNAP-25 (1–197) constructs, were homogenized, followed by membrane extraction, yielding the cytosolic and the soluble membrane fractions, which were analyzed by western blotting. While membrane protein Sortilin was enriched in membrane fractions, GAPDH was not present in the same fractions, suggesting successful fractionation. Data suggest that SNAP-25 (1–197) is localized on the membrane similar to WT SNAP-25, regardless of PMA treatments (Fig 4A). These findings are consistent with previous work demonstrating that BoNT/A-cleaved SNAP-25 is localized on the plasma membrane [27]. However, importantly, when there are roughly equal amounts of WT SNAP-25 and SNAP-25 (1–197) on the membrane, Ser187 phosphorylation levels of SNAP-25 (1–197) fragments are greater compared to that of WT SNAP-25, which become more obvious upon PMA treatments (Fig 4A, bar graph). We further evaluated the membrane localization of WT SNAP-25 and SNAP-25 (1–197) in PC12 cells, which endogenously express WT SNAP-25. We transfected the BoNT/A LC plasmid into PC12 cells and sought to achieve a minimal SNAP-25 cleavage, yielding low levels of SNAP-25 (1–197), as compared to large fractions of WT SNAP-25, to try to mimic conditions in which a tiny fraction of cleaved SNAP-25 leads to near complete synaptic inhibition [10,13–17]. Enrichment of membrane proteins syntaxin-1A and absence of GAPDH in membrane fractions suggest successful fractionation (Fig 4B). Our data demonstrated that SNAP-25 (1–197) is present in membrane fractions in both PMA-treated and untreated conditions, despite comparatively less SNAP-25 (1–197) as compared to WT SNAP-25 in cells (Fig 4B, total lysate conditions). Similar to the results obtained in HEK293 cells (Fig 4A), the data demonstrate that membrane-localized WT SNAP-25 and SNAP-25 (1–197) are both Ser187 phosphorylated; however, Ser187 phosphorylation levels of SNAP-25 (1–197) fragments are greater compared to that of WT SNAP-25 (Fig 4B). We have also included non-soluble fractions in our analyses to measure WT SNAP-25 and SNAP-25 (1–197) levels remaining in the insoluble fractions during membrane extraction. Although most SNAP-25 present in a cell is localized on the cell membrane, SNAP-25 can also be present in recycling endosomes and the trans-Golgi network compartments [53]. For example, it has been shown that about 20% of SNAP-25 is located in a perinuclear recycling endosome-trans-Golgi network [54]. Our data demonstrated that WT SNAP-25 was more abundant than SNAP-25 (1–197) in the insoluble fractions. Overall, our data suggest that both in HEK293 and PC12 cells, SNAP-25 (1–197) is localized on the membrane, and the Ser187 phosphorylation of the membrane-localized SNAP-25 (1–197) is greater than that of WT SNAP-25.

### BoNT/A-cleaved SNAP-25 is detected in cultures longer than BoNT/A LC

A large body of literature concluded that long-lasting biological effects of BoNT/A depend on the stability of the BoNT/A enzymatic component [9]. For example, it has been suggested that BoNT/A LC can be biologically active for more than 10 months in culture [55] and *in vivo* rodent brains [56]. However, these studies concluded about the toxin’s presence using SNAP-25 cleavage as the readout, which does not distinguish whether the toxin’s enzymatic component was indeed present after many months, or previously cleaved SNAP-25, SNAP-25 (1–197), survived. Although the longer half-life of BoNT/A compared to other serotypes has been well documented [25,57–60], whether the duration of the stability extends to many months is unclear. To characterize the temporal lifetime of cellular BoNT/A LC and SNAP-25 (1–197) proteins, we designed the experiments based on previously characterized mammalian cell line plasmid decay lifetimes post-transient transfection [61,62]. It was demonstrated that the half-life of 4 µg plasmids transfected to HEK293 cells using Lipofectamine 2000 is about 14 hours between days 1 and 2 post-transfection [61]. We transiently transfected 0.5 µg plasmids encoding YFP-tagged BoNT/A LC, WT-SNAP-25, or SNAP-25 (1–197) to HEK293 cells using Turbofect, collected cell lysates at various time points, and performed western blot analyses. BoNT/A LC was not detected after 5 days, while both WT SNAP-25 and SNAP-25 (1–197) proteins were still present in cultures at the latest time point tested (Day 20) (Fig 4C). To further explore this phenomenon, we transiently transfected PC12 cells, which endogenously express SNAP-25, with BoNT/A LC to generate BoNT/A-cleaved SNAP-25, collected samples at various time points, and detected BoNT/A LC and SNAP-25 (1–197) via western blots. Our results demonstrated that SNAP-25 (1–197), although at lower levels, was still detectable on Day 23, and BoNT/A LC protein was not detected after Day 10 (Fig 4D). Collectively, these data suggest that SNAP-25 (1–197) persists longer than BoNT/A LC in cells, which may have a role in the prolonged effects of BoNT/A-mediated poisoning (further discussed in the Discussion section).

### BoNT/A-cleaved SNAP-25 fragment binds to syntaxin-1A, and the interaction is enhanced by Ser187 phosphorylation

**Fig 5:**
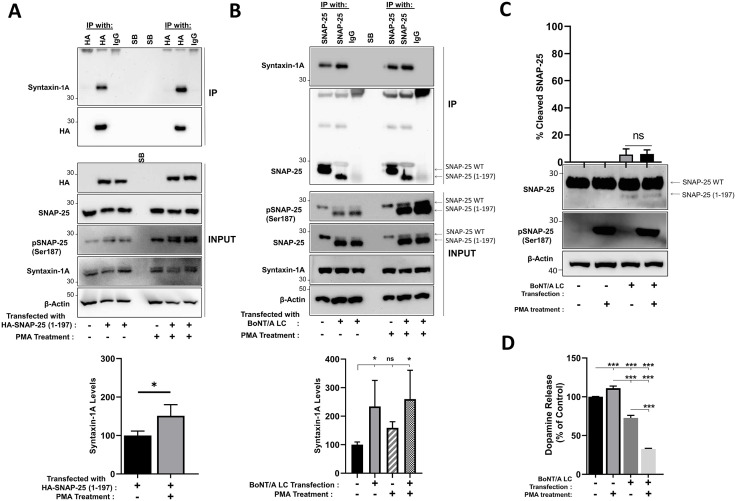
SNAP-25 (1-197) binds to syntaxin-1A, and the interaction is enhanced by Ser187 phosphorylation. **(A)** PC12 cells were transfected with an HA-tagged SNAP-25 (1-197) plasmid and then treated with 1 µM PMA (a total of 1.5 hours). Immunoprecipitation (IP) was conducted using rabbit polyclonal anti-HA antibodies or normal rabbit IgGs. Input and IP samples were subjected to immunoblotting to detect the levels of the indicated markers. Blots are representative of 4 independent experiments. Syntaxin-1A band intensities were normalized to HA levels that were reprobed on the same IP blots. Syntaxin-1A intensity in the PMA-treated HA immunoprecipitates was compared to non-PMA-treated conditions. **(B)** PC12 cells were transfected with the BoNT/A LC plasmid, and 1 µM PMA treatment was conducted in the indicated samples (a total of 1.5 hours). Immunoprecipitation was carried out using mouse monoclonal anti-SNAP-25 or normal mouse IgGs. Western blotting was conducted to detect the levels of the indicated markers in both input and IP samples. Representative blots of 4 independent experiments are shown. Syntaxin-1A intensity normalized to SNAP-25 levels in each IP blot was quantified. ns: non-significant; *: P ≤ 0.05; **: P ≤ 0.01; ***: P ≤ 0.001. **(C and D)** PMA treatment further decreases neurotransmitter release when a low level of SNAP-25 cleavage is present. PC12 cells were transfected with 1 µg YFP-tagged BoNT/A LC and treated with and without 1 µM PMA. Dopamine release was stimulated by high K+ solution treatment, and supernatant was analyzed via a triple quadruple LC-MS/MS system (Waters TQ-S Micro triple quadruple LC-MS/MS). **(C)** Cell lysates were collected following the high K+ solution treatment and subjected to immunoblotting. The percentage of cleaved SNAP-25 was calculated in each condition and presented in the bar graph. A separate, identical gel was run to assess PMA-mediated SNAP-25 phosphorylation. β-Actin was used as a loading control. **(D)** Mass spectrometry analysis of dopamine release from PC12 cells. The bar graph demonstrates the percentage of dopamine release relative to vehicle (DMSO) control. Data are representative of 3 independent experiments, and results are presented as mean ± SD. *: P ≤ 0.05; **: P ≤ 0.01; ***: P ≤ 0.001.

SNARE proteins are typically maintained in inactive conformations in cells to prevent untimely neurotransmitter release [63]. Switching to active conformation requires, as a first step, physical interaction of syntaxin-1A with SNAP-25, allowing SNARE motif associations between these two proteins [64]. PMA-induced enhancement of SNAP-25 phosphorylation has been demonstrated to increase SNARE protein interactions as determined by immunoprecipitation [40]. Therefore, we have evaluated whether SNAP-25 (1–197) interacts with syntaxin-1A directly and whether Ser187 phosphorylation of the fragment impacts any such potential interaction. First, we transfected HA-tagged SNAP-25 (1–197) into PC12 cells, which endogenously express WT-SNAP-25 and syntaxin-1A, and conducted immunoprecipitation with anti-HA or IgG control antibodies. We PMA-treated cells to induce Ser187 phosphorylation of SNAP-25. We observed that SNAP-25 (1–197) interacts with syntaxin-1A, and importantly, PMA treatment enhances the SNAP-25 (1–197)-syntaxin-1A interaction (Fig 5A). Our results are consistent with previous literature suggesting that BoNT/A-cleaved SNAP-25 can interact with syntaxin-1A, and SNAP-25 phosphorylation at Ser187, only reported phosphorylation site in the SNARE domain at the C-terminus [65], enhances SNAP-25-syntaxin-1A interaction [38,40,43]. To further test our findings, we have transfected BoNT/A LC plasmid into PC12 cells to achieve SNAP-25 cleavage, conducted immunoprecipitation using monoclonal anti-SNAP-25 antibodies, and explored syntaxin-1A pull-down with western blotting (Fig 5B). This approach is challenging because, while syntaxin-1A (~33kDa) can be easily detected with specific antibodies in western blot analyses, light chain IgG band (~25 kDa) resulting from the IP can potentially hinder the SNAP-25 signal (~25 kDa) when the same blots were reprobed for SNAP-25. However, previous literature demonstrated that the SNAP-25 band can be separated from the IgG light chain band when immunoprecipitated samples are subjected to western blotting [66]. Similarly, we resolved the SNAP-25 signal, which was reprobed to each syntaxin-1A blot, and quantified syntaxin-1A pull down in each condition. The results suggested that SNAP-25 (1–197) interacts with syntaxin-1A, and that its level is higher than that of WT SNAP-25 -syntaxin-1A interaction. Overall, our data indicate that higher Ser187 phosphorylation provides a competitive advantage for SNAP-25 (1–197) over WT SNAP-25 for syntaxin-1A interaction and thereby SNARE complex participation.

### PMA treatment exacerbates neurotransmitter release reduction under conditions of low-level SNAP-25 cleavage

To investigate whether enhanced phosphorylation of SNAP-25 influences neurotransmitter release, we conducted mass spectrometry-based analyses to analyze neurotransmitter release, particularly in conditions where only minimal SNAP-25 cleavage was observed. As shown in Fig 5C a very low level of SNAP-25 cleavage was achieved, and we sought to measure dopamine levels released to the culture, as prior studies suggested that dopamine detection is more efficient and reliable in PC12 cells [67]. Our analyses revealed that a low level of SNAP-25 cleavage (~5%) led to a substantial reduction (~27%) in dopamine release (Fig 5D). This observation is consistent with previous reports demonstrating that even a small fraction of SNAP-25 (1–197) is sufficient to markedly inhibit neurotransmission [10,13–18], although the extent of inhibition may vary depending on the cell type and the specific assay employed. Importantly, PMA treatment further decreased neurotransmitter release when low levels of SNAP-25 cleavage were present (Fig 5C and 5D). In contrast, in the absence of SNAP-25 cleavage in normal cells, PMA treatment resulted in an aproximate 11% increase in dopamine release (Fig 5D). Although the precise mechanism by which PMA affects neuroexocytosis remains unclear, our observations are in agreement with earlier studies in PC12 cells demonstrating that 1µM PMA treatment enhances KCl-stimulated neuroexocytosis [68,69]. For example, PMA-mediated increases in neurotransmitter release ranging from approximately 20–40% have been reported in PC12 cells under high-K^+^ solution stimulation [40,51]. Taken together, our results suggest that the further reduction in dopamine release following PMA treatment under conditions of minimal SNAP-25 cleavage may be attributed to PMA-mediated phosphorylation of SNAP-25 (1–197). This provides a mechanistic link between Ser187 phosphorylation of SNAP-25 (1–197) and its functional contribution to the dominant negative effect on neuroexocytosis.

To investigate whether SNAP-25 phosphorylation at Ser187 affects the thermal stability of SNARE complexes, we conducted membrane extraction analyses similar to those shown in Fig 4 and assessed SNARE complexes under boiled and non-boiled conditions, upon PMA treatment, using western blotting (S2 Fig). Successful PMA treatment and membrane extraction efficiency were confirmed (S2A Fig), and then total lysates, along with the soluble membrane and cytosolic fractions, were subjected to western blotting following thermal treatment at 95°C for 5 minutes or under non-boiled conditions. The results indicate that SNARE complexes formed in the presence of PMA, leading to enhanced SNAP-25 Ser187 phosphorylation, are not thermally stable (S2B Fig).

### Molecular Modeling suggests that Ser187 phosphorylation changes the local dynamics and electrostatic signature of the SNARE complex while not affecting the overall binding ability of SNAP-25 to the complex

**Fig 6:**
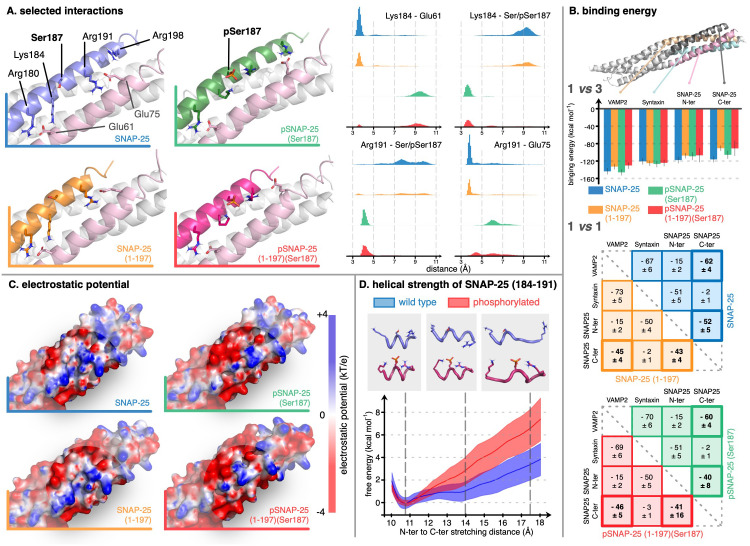
Molecular modeling suggests phosphorylation of Ser187 does not impact SNAP-25-SNARE complex binding, but changes its local dynamics and electrostatic signature. The four panels summarize the results of molecular modeling on the SNARE complexes. **(A)** Focus on the local changes induced in the interaction pattern around Ser187 of SNAP-25 and the distribution of relevant inter-side chains distances along the simulations. **(B)** Binding energy is calculated for each fragment against the other three in the complex (1 vs 3) and for each pair of fragments individually (1 vs 1). The latter is given in a matrix representation with an averaged value and corresponding standard deviation for each considered SNARE complex. The residues included in the calculation of binding energy are highlighted in the upper part of the panel. **(C)** Focus on the local changes induced in the electrostatic potential around Ser187 of SNAP-25. **(D)** Free energy profiles for the unwinding process of a small peptide, representative of the sequence around Ser187 in SNAP-25.

Finally, we performed molecular dynamics simulations of four variants of the SNAP-25 in the SNARE complex: WT SNAP-25, SNAP-25 (1–197), pSNAP-25 (Ser187), and pSNAP-25 (1–197) (Ser187) (i.e., BoNT/A-cleaved and Ser187-phosphorylated). It is important to note that our molecular modeling addressed the ternary SNARE complex and not earlier intermediates such as the syntaxin–SNAP-25 binary complex. In the absence of a high-resolution structure for this intermediate, modeling its behavior upon phosphorylation would require speculative assumptions and was therefore not attempted here. Our results are summarized in Fig 6. Overall, our simulations show no significant effect at the scale of the whole complex upon cleavage and/or phosphorylation. The backbone RMSD time series provided in S1 Fig indicate that the structure of the complex remains the same in all cases and is very stable during the dynamics. The value of RMSD and the amplitude of fluctuations are slightly reduced upon phosphorylation. A careful look at the atomic scale reveals a few critical local changes in interaction patterns around the phosphorylation site, Ser187. Fig 6A shows a close-up view of the complex in the region around Ser187 of SNAP-25. The two helices in the foreground are the N- and C-terminal domains of SNAP-25. In the absence of phosphorylation, three positively charged side chains of C-ter SNAP-25 (Arg180, Lys184, and Arg191) interact with two glutamates of N-ter SNAP-25 (Glu61 and Glu75). These interactions are very stable along the dynamics as demonstrated by the distance distributions in the right panel of Fig 6A. Ser187 phosphorylation of SNAP-25 changes this interaction pattern as Lys184 and Arg191 now engage in intra-helix interaction with pSer187 (phosphorylated Ser187), thus breaking some links with the N-terminal part of SNAP-25. This observation is also valid in the complex with pSNAP-25 (1–197) (Ser187) but the interaction partner of Arg191 oscillates between Glu75 and pSer187. In Fig 6B, we observe that the interaction changes have an effect on the binding energy between fragments of the SNARE complex. The binding of syntaxin-1A to the other three helices is not affected by any change in C-ter SNAP-25, which is understandable considering that these two fragments do not interact directly with each other. VAMP2 binding is only affected by cleavage. About 15 kcal mol^-1^ of binding strength is lost when the C-ter part of SNAP-25 is cleaved and phosphorylation has no effect on VAMP2 since Ser187 points towards the N-terminal part of SNAP-25. In the 1 vs 1 matrix representation of binding energy, we show the decomposition of the overall binding of each fragment to the others. The binding of VAMP2 to C-ter SNAP-25 is about -60 kcal mol^-1^ regardless of the phosphorylation state of Ser187, while it becomes about -45 kcal mol^-1^ upon cleavage. Comparing the binding of N-ter and C-ter SNAP-25 in the wild-type and cleaved complexes, we observe a similar trend, with about 10 kcal mol^-1^ decrease in binding strength. Comparing SNAP-25 and pSNAP-25 (Ser187) shows that phosphorylation has the same 10 kcal mol^-1^ effect as cleavage does. Interestingly, the two effects are not cumulative as SNAP-25 and pSNAP-25 (1–197)(Ser187) show only a 10 kcal mol^-1^ difference in binding energy between the two SNARE domains of SNAP-25. One significant difference between all variants considered in this work and pSNAP-25 (1–197) (Ser187) is in the magnitude of the standard deviation for the binding energy between the two SNAP-25 helices. While it is only a few units for all pairs of fragments, it reaches 16 kcal mol^-1^ in the latter case, indicating that the complex with pSNAP-25 (1–197)(Ser187) shows a greater flexibility in its binding pattern compared to the other three. This is in line with the above report that Arg191 can change binding partners between Glu75 and pSer187 only in the case of a cleaved and phosphorylated SNAP-25. Comparing with the backbone RMSD time series (S1 Fig) confirms that this fluctuation is only due to side chains and not to a change in the overall structure of the complex. The change in interaction pattern induced by phosphorylation also affects the electrostatic potential on the surface in the region around Ser187, as demonstrated in Fig 6C. Therein, one can see that the two non-phosphorylated complexes have the same electrostatic signature, with a neutral or positive and rather rough surface around Ser187, while the pattern changes upon phosphorylation. In the latter case, the holes are closed and the neutral and positive regions disappear to give place to a more negative surface. Finally, we investigated the effect of phosphorylation and the concurrent formation of strong interactions between pSer187 and neighboring residues on the stability of the helical secondary structure. As reported above, Lys184 and Arg191 only engage in interactions with Ser187 when the latter is phosphorylated. We simulated a short peptide reproducing the direct environment of Ser187 and measured the helical strength via umbrella sampling. Our strategy follows a similar approach to that of Park et al. [70]. When stretching the wild-type peptide (with canonical Ser187), the free energy profile is fairly flat up to a distance of 14.0 Å between the two end α-carbon atoms. When Ser187 is phosphorylated, however, its strong interaction with Lys184 and Arg191 significantly stabilizes the helical structure as the energy rises sharply when the distance is moved away from its minimum at 10.8 Å. The phosphorylation of Ser187 appears to significantly increase the helical strength of this portion of the sequence in C-ter SNAP-25.

## Discussion

**Fig 7:**
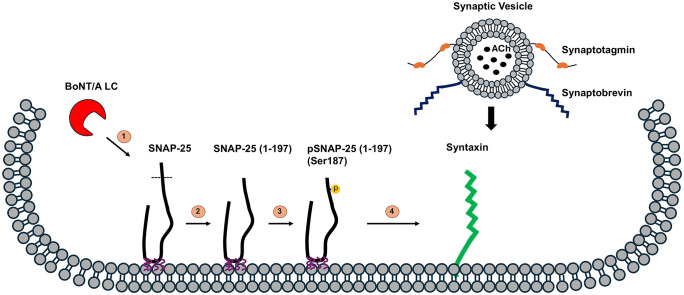
Proposed model of the mechanism underlying the dominant negative effect of BoNT/A cleaved SNAP-25 on neurotransmitter release. (1) BoNT/A LC cleaves SNAP-25; (2) Cleaved fragment, SNAP-25 (1-197), stays on the membrane; (3) SNAP-25 (1-197) gets efficiently phosphorylated at Ser187 residue; (4) Ser187 phosphorylation provides a competitive advantage for SNAP-25 (1-197) for syntaxin-1A binding over WT SNAP-25, which can lead to a dominant-negative effect.

This work provides mechanistic insights into how BoNT/A-cleaved SNAP-25 has a dominant-negative effect on neuroexocytosis. SNAP-25 (1–197) has long been hypothesized to inhibit neurotransmitter release by competing with WT SNAP-25 for SNARE complexes [10–12]. This hypothesis is supported by findings from multiple groups that BoNT/A-mediated truncation of only a very small fraction of the total SNAP-25 pool is sufficient to significantly inhibit neuroexocytosis. On the contrary, BoNT/E-mediated cleavage of SNAP-25 levels correlates well with the related inhibition of exocytosis. One hypothesis for this serotype difference is that BoNT/E-mediated cleavage products may not compete with WT SNAP-25 to produce a dominant-negative effect. However, the molecular mechanisms underlying the potential dominant-negative roles of cleaved fragments were unknown. The C-terminal cleaved part of the SNAP-25 is important for proper neuroexocytosis [19,20], as BoNT/A-truncated SNAP-25, SNAP-25 (1–197), inhibits neuroexocytosis [24]. It has been shown that BoNT/E-mediated SNAP-25 cleavage leads to the dissociation of syntaxin/SNAP-25 interaction, resulting in SNAP-25 dissociating from the plasma membrane [71]. However, SNAP-25 (1–197) localizes to the membrane [27] (Fig 4), and the interaction between SNAP-25 and syntaxin-1A persists even after BoNT/A-mediated SNAP-25 cleavage and still forms stable SNARE complexes [28,29]. Considering the differences between BoNT/A and/E-generated SNAP-25 fragments, the SNAP-25 sequence between Ile181 and Gln197, which is present on BoNT/A-cleaved SNAP-25 but not in that of BoNT/E, is likely crucial for SNARE complex stability [29]. This region includes only one reported phosphorylation residue (Ser 187). Our findings demonstrate that Ser187 is heavily phosphorylated in SNAP-25 (1–197) fragments (Figs 1–4), whereas SNAP-25 (1–180) does not contain the residue. Importantly, Ser187-phosphorylated SNAP-25 (1–197) fragments are membrane-associated (Fig 4), show enhanced co-immunoprecipitation with syntaxin-1A (Fig 5A and 5B), and form stable SNARE complexes (Fig 6). While our modeling did not support increased thermodynamic binding affinity, consistent with the spatial separation between Ser187 and the syntaxin interface, the experimental data suggest that phosphorylation may alter early binding kinetics or complex dynamics. Together, these findings support the idea that SNAP-25 (1–197), once phosphorylated, acts as a dominant-negative form not by preventing SNARE complex formation, but by creating functionally inert complexes that outcompete WT SNAP-25. As a result, neuroexocytosis is severely compromised, as SNAP-25 (1–197) participating SNAREs cannot complete neurotransmitter release [24] (Fig 7). These results align with previous findings that BoNT/A-mediated SNAP-25 cleavage does not affect SNARE formation but leads to vesicle docking defects at the plasma membrane, resulting in very drastic inhibition of vesicle fusion [17].

Previous research demonstrated that BoNT/A-mediated inhibition of neuroexocytosis lasts the longest compared to other human botulism-causing serotypes [58,72,73], though the underlying molecular mechanisms are not well understood. For example, BoNT/A intoxication can exhibit effects for several months, while the biological effects of BoNT/E last only a few weeks in humans [25,57,58]. A commonly accepted hypothesis regarding the long-lasting effects of BoNT/A is that the duration of the BoNT/A-mediated paralysis solely depends on the cytosolic lifetime of the BoNT/A LC [9]. This hypothesis was supported by findings that BoNT/A LC avoids proteasomal degradation, despite efficient ubiquitination, via the dominant function of VCIP135 deubiquitinase, while BoNT/E LC is efficiently ubiquitinated and degraded in cells [59,60]. Nonetheless, whether BoNT/A LC can evade degradation in cells for many months is unclear. BoNT/A-cleaved SNAP-25 has been previously detected in primary rat spinal cord neurons for at least 10 months [55]. Similarly, cleaved SNAP-25 was shown to be detectable after 12 months in rat brains injected with 1 ng of BoNT/A [56]. However, the readout in these studies was SNAP-25 truncation, and it is unclear whether the toxin remained intact and active for such a long time or whether previously cleaved SNAP-25 survived. A second hypothesis suggests that a small fraction of total SNAP-25 may be actively involved in neuroexocytosis, and cleavage of such a pool by BoNT/A inhibits neurotransmission. However, this hypothesis does not explain the correlation between BoNT/E-mediated cleavage and exocytosis inhibition, as all the SNAP-25 appears accessible to BoNT/E [13,14]. A third hypothesis suggests that the persistence of BoNT/A-cleaved SNAP-25, independent from the continuous presence of BoNT/A, occurs through a dominant-negative mechanism of action [10–12]. SNAP-25 (1–197) has been observed to be present in cultures for more than multiple months [55,72], and SNAP-25 (1–197) is more stable than SNAP-25 (1–180) [10]. Our findings from this study support this hypothesis and suggest that Ser187-phosphorylated SNAP-25 (1–197) may interfere with normal functions of WT SNAP-25 in a dominant-negative manner, and that this interference may be critical for the observed prolonged effects of BoNT/A.

While our work here mainly addresses the intra-complex effect of SNAP-25 (1–197), it is important to recognize that alternative or concurrent mechanisms may exist. Specifically, the altered electrostatics and conformational dynamics introduced by Ser187 phosphorylation of SNAP-25(1–197) may affect interactions beyond the core SNARE complex. In particular, such modifications could lead to sequesteration of SNARE-associated proteins or interfere with accessory factors involved in vesicle docking and priming. Such hetero-complex disruptions may amplify the functional blockade and help explain the disproportionate impact of a relatively small pool of cleaved SNAP-25 molecules on neurotransmitter release. A radial SNARE supercomplex model, emphasizing the spatial arrangement of SNARE motifs to form a larger octameric ring, has been proposed with experimental evidence in Drosophila [11,12,74], which may be the potential underlying mechanism of the dominant negative effect of SNAP-25 (1–197). However, direct experimental evidence demonstrating the incorporation of cleaved SNAP-25 acting as a dominant-negative factor within an octomeric SNARE super complex in mammals is currently lacking [75]. Although there is no consensus on the precise number of SNAREs per vesicle required for facilitating neurotransmission, previous work suggested that as few as one to three SNARE complexes can suffice for membrane fusion [76–82]. Nonetheless, higher-order assemblies, including hexameric SNARE complexes, have also been reported in mammalian neuroexocytosis [83–85], which can potentially form symmetric, ring-like structures [86,87]. This raises the possibility that cleaved SNAP-25 may impair the cooperative function of multiple SNARE complexes. Importantly, this mechanism is not mutually exclusive with impairment at the level of individual complexes, and both processes may co-exist and jointly account for the observed functional consequences. Future work should elucidate whether pSNAP (1–197) (Ser187), when incorporated into individual SNARE complexes, perturbs the organization and dynamics of higher-order SNARE assemblies.

We determined that BoNT/A-cleaved SNAP-25 survives longer than the BoNT/A LC protein in cells. Long-lasting BoNT/A effects have been primarily correlated to the long half-life of the BoNT/A LC itself; however, time course experiments revealed that BoNT/A-cleaved SNAP-25 was detectable in cells at least 23 days after (the latest time point tested in PC12 cells), while BoNT/A LC was not detected after 10 days (Fig 4C and 4D). Previous work suggested that the SNAP-25 protein has a relatively short half-life. For example, SNAP-25 half-life was calculated to be about 10 hours in PC12 cells [88] and 35 hours in *in vitro*-maturated rat primary neurons [89]. In the above studies, despite a substantial reduction, a detectable SNAP-25 level was still present in the latest time point tested. This observation is important, as previously discussed, because a fraction of SNAP-25 (1–197) can sufficiently inhibit exocytosis. It is important to note that BoNT/A LC versus SNAP-25 (1–197) longevity experiments in this study have several methodological limitations. First, the approach is based on overexpression of both proteins, without normalization for the initial number of molecules expressed, which may affect time-dependent comparisons of protein degradation. Second, the use of different antibodies for BoNT/A LC (GFP antibody was utilized) and SNAP-25 (1–197) introduces potential bias due to differences in epitope affinity and detection sensitivity. Third, although the relative levels of the proteins appear to be gone, BoNT/A LC enzyme may remain active below the detection threshold of western blotting, continuing to cleave SNAP-25 even in the apparent absence of detectable enzyme. Fourth, the BoNT/A LC includes a tag, which could potentially affect its intracellular BoNT/A LC degradation kinetics. Consequently, the persistent SNAP-25 (1–197) signal may reflect a combination of LC/A persistence and the stability of SNAP-25 (1–197) fragments, rather than the longevity of SNAP-25 (1–197) alone. These limitations highlight the need for future studies to further explore SNAP-25 (1–197) longevity in comparison to BoNT/A LC and WT SNAP-25. Taken together, based on our findings, we propose that SNAP-25 persistence may reflect both BoNT/A LC stability and the longevity of cleaved SNAP-25 fragments.

A number of modeling studies of the SNARE complex have been published over the past few years [90–94], and some of them were summarized in short reviews [95,96]. The remarkable work by Sharma and Lindau, involving coarse grain models, revealed great details of the fusion process [93]. In particular, the authors highlighted that simulations with SNAP-25 (1–197) (designated SNAP-25Δ9 in the article) allow stalk formation but inhibit further steps of the fusion mechanism. Similarly, mutation of the C-terminal region of VAMP2 by the addition of two lysines (named syb2-KK in the article) produces the same effect as cleaving SNAP-25, yet with a different mechanism. In the latter case, the added positively charged side chains strongly interact with the membrane’s negatively charged head groups, which effectively stabilizes the complex at the surface of the membrane. It is worth noting that while these two mutations consist of fairly small, local changes near the linker region of the SNARE complex, they have a dramatic impact on the biological process in which the complex is involved. Similarly, the local changes observed in our simulations upon phosphorylation of Ser187 are expected to have an impact on the role of the SNARE complex. As demonstrated in various models cited above, the C-terminal part of the two helices of SNAP-25 in the SNARE complex is in close contact with negatively charged heads of phospholipids at the membrane surface in the early stage of the fusion mechanism. The drastic change in electrostatic signature from neutral/positive to mostly negative after phosphorylation of Ser187 may affect the fusion process. It is unclear, however, if the increased rigidity of the helical structure around the phosphorylation site, as our models suggest, impacts the zippering mechanism in one way or another. Nevertheless, considering how tightly controlled the fusion process is, it is likely that any deviation from the optimal behavior of the SNARE complex can block exocytosis. Overall, our models of the full SNARE complex illustrate that its formation is not impaired by cleavage and/or phosphorylation, and the resulting structure appears similarly stable, which is consistent with previous studies [28,29]. However, phosphorylation induces local changes in the conformational dynamics and electrostatic profile of the C-terminal region near the membrane interface, which may significantly disrupt downstream steps in the fusion mechanism. It has been suggested that cleavage of SNAP-25 does not prevent SNARE complex formation, but cleavage might impair the fusion event [97]. Combining our experimental and computational findings, we can infer that cleavage not only directly impacts fusion but also promotes Ser187 phosphorylation within SNAP-25, which in turn affects the function of the SNARE complex without preventing its formation. These results suggest that further studies on the role of Ser187 phosphorylation will provide further impactful insights into SNAP-25 and BoNT/A function. Replicating the fusion models by Sharma and Lindau [93] with phosphorylated Ser187 would likely be particularly valuable. While our data support a mechanism in which functionally inert SNARE complexes block productive usage, we also acknowledge that SNAP-25 (1–197), particularly when phosphorylated at Ser187, could disrupt additional protein-protein interactions involved in SNARE regulation or recycling. Such interference, whether through sequestration or altered docking dynamics, may further reinforce the blockade and contribute to long-lasting functional inhibition. Although these effects were not directly addressed in our modeling, they represent promising avenues for future investigation.

Previous studies have shown that Ser187 phosphorylation of SNAP-25 enhances secretion in various cell lines [32,37–40]. Furthermore, investigations using a genetically modified mouse model in which Ser187 was substituted with alanine suggested that this phosphorylation site plays functional roles in vivo, as evidenced by defects in serotonin and dopamine release [33], impairments in working memory [34], alterations in basal synaptic transmission [35], and susceptibility to epilepsy-related seizures [36]. However, these findings have been questioned, with some studies proposing that Ser187 phosphorylation of SNAP-25 is not crucial for neurotransmitter release [41,42]. Our results in this work support prior in vitro and in vivo studies that Ser187 phosphorylation of SNAP-25 has functional significance (Fig 5C and 5D). Multiple groups have established that the Ser187 phosphorylation enhances syntaxin/SNAP-25 interaction, leading to enhanced SNARE complex assembly [38,40,43]. Our co-immunoprecipitation data (Fig 5A and 5B) support increased association between Ser187 phosphorylated SNAP-25 (1–197) and syntaxin-1A in cells. This enhanced association may reflect either increased intrinsic affinity or greater kinetic stability of the complex. Co-immunoprecipitation experiments cannot distinguish between these possibilities. Our molecular modeling, performed on the full SNARE complex, did not support a direct effect of Ser187 phosphorylation on complex thermodynamic stability (Fig 6), consistent with the location of Ser187 being distant from the syntaxin interface. This suggests that phosphorylation may impact early complex formation or post-assembly steps, rather than thermodynamic stabilization.

Another important finding of this work is that the increased SNAP-25 phosphorylation upon BoNT-mediated cleavage is Ser187-specific. Ser187 and Thr138 are the only phosphorylation events with functional consequences reported for SNAP-25. Previous research suggested that Thr138 negatively regulates SNARE formation by decreasing SNAP-25 binding to syntaxin [38,40,44], which is opposite to the effects of Ser187 [38,40,43]. Our work suggests that BoNTA-mediated removal of 9 amino acids from the SNAP-25 C-terminus may expose Ser187 for efficient phosphorylation by PKC. Given that Thr138 is a crucial SNAP-25 phosphorylation residue, future work elucidating the role of Thr138 phosphorylation in SNARE regulation may further inform how exocytosis is regulated in the presence or absence of BoNT intoxication. Computationally, while we did not model the role of Thr138 phosphorylation in this work, a visual inspection of the crystal structure with PDB-ID 1SCF is nonetheless informative when interpreted in light of our findings for Ser187. Thr138 is located at the C-terminal end of the linker loop that bridges the N- and C-terminal regions of SNAP-25 and is involved in a turn secondary structure [92]. Similarly to Ser187, Thr138 is surrounded by positively charged groups in the SNAP-25 sequence, i.e., Arg135, Arg136, and Arg142. Thr138 phosphorylation could be inferred to have a similar impact to Ser187 phosphorylation, i.e., forcing interactions between positively charged side chains and the phosphorylated hydroxy group. In the case of Ser187, we showed that it stabilizes the helical structure, which is necessary for forming the SNARE complex. However, disrupting the turn motif around Thr138 could prevent proper positioning and eventually the binding of the N- and C-terminal parts of SNAP-25 in the SNARE complex. Modeling this hypothesis should, however, be the focus of a dedicated study.

In summary, this study provides mechanistic insights into the dominant-negative effect of BoNT/A-cleaved SNAP-25 on neurotransmitter release. Our work has therapeutic implications for countering BoNT/A intoxication through at least two distinct mechanisms. First, targeting SNAP-25 Ser187 phosphorylation may facilitate the discovery of novel anti-BoNT/A therapeutics, which can be achieved through either kinase inhibitors or phosphatase activators. Compared to phosphatase modulators, there have been significant advancements in the development of kinase inhibitors. There are 88 different FDA-approved kinase inhibitors targeting more than 20 different kinases [98], although the majority of which are approved for oncological conditions, these inhibitors hold potential for other diseases, including those affecting the human nervous system. However, within the BoNT intoxication context, this approach would require targeted delivery to motor neuron end terminals to reach a therapeutic dose, as global inhibition of kinases or activation of phosphatases may lead to widespread off-target effects, emphasizing the need for spatially controlled delivery methodologies. The second challenge is that the selective reduction in the SNAP-25 (1–197), without disturbing WT SNAP-25 phosphorylation dynamics, is required to counter BoNT/A intoxication without disturbing normal SNAP-25 function in exocytosis. Secondly, increasing the turnover of SNAP-25 (1–197) regardless of its phosphorylation state might have a therapeutic value, as our work highlights the importance of the clearance of SNAP-25 (1–197) regardless of its phosphorylation state. Targeted degradation of SNAP-25 (1–197) in BoNT/A intoxicated neurons could mitigate the effects of the toxin without affecting WT SNAP-25 function. Given that multiple research groups have already developed antibodies that selectively detect SNAP-25 (1–197) but not WT SNAP-25 [99–105], this highlights the potential feasibility of selective degraders to specifically eliminate the cleaved fragment. Furthermore, advances in Proteolysis-targeting chimeras (PROTAC)-mediated degradation of BoNT/A [106] and neuronal delivery methodologies against the toxin [107,108] may facilitate the development of selective SNAP-25 degraders. Since SNAP-25 (1–197) remains anchored to the cytosolic leaflet of the plasma membrane, which is a barrier for applying classical PROTACs, alternative intracellular degradation strategies such as AUTACs, ATTECs, and Trim-Away may represent more suitable approaches, as they can target cytosolic-facing proteins via autophagy- or proteosome-mediated pathways [109,110]. Targeting the SNAP-25 (1–197) fragment in this way could restore normal neuroexocytosis without compromising the essential functions of intact SNAP-25. Regulating the degradation kinetics of SNAP-25 (1–197) that may enable more precise control over BoNT intoxication can also offer potential benefits for clinical applications.

## Materials and methods

### Mouse and human embryonic stem cell (ESC) culture and their directed differentiation to motor neurons

Mouse ESC line (HBG3) was cultured and differentiated into motor neurons based on our previously established protocols [111–113]. H9 human ESC line (WA09) was purchased from WiCell Research Institute, University of Wisconsin-Madison, and was cultured and differentiated into motor neurons to use in BoNT studies based on our previous studies [114,115].

### Plasmids

Mammalian expression clones of both WT SNAP-25b and SNAP-25b (1–197) were generated by the Protein Expression Laboratory, Advanced Technology Program, SAIC-Frederick. All plasmids were subcloned using the Gateway system into new vectors. Human cDNAs of WT SNAP-25b, SNAP-25b (1–197), HA-SNAP-25b (1–197), and the following base pair mutations, SNAP-25 S187A and SNAP-25 S187D, were generated and cloned into the mammalian expression vector pcDNA3.1 + neo (Thermo Fisher) for protein expression studies. YFP-tagged BoNT/A LC plasmid was a kind gift from Dr. Yien Che Tsai (NCI-Frederick, NIH, USA).

### Cell culture, transfections, and membrane extraction experiments

PC12 cells were cultured on gelatin and collagen-coated plates in a growth medium including High-Glucose DMEM, 5% FBS, 10% Heat-inactivated Horse Serum, and 1%Pen-Strep. For the cultivation of the HEK293 cell line, the media included High-Glucose DMEM, 10% FBS, 1%GlutaMAX, and 1% Pen-Strep. SH-SY5Y cells were cultivated in a growth medium containing advanced DMEM-F12, 10% FBS, and 1% Pen-Strep. All three cell lines were incubated at 37°C, with 5% CO_2_. Transient transfections in HEK293 cells were performed with Turbofect (Thermo Fisher, R0533) or X-tremeGENE 9 DNA (Roche, 06365787001) transfection reagents using 1 μg plasmid DNA unless otherwise stated. Phorbol 12-myristate 13-acetate (PMA) (Sigma-Aldrich, P8139) was utilized as a PKC activator, and the treatment conditions are described within the figure legends. In the protein stability experiments demonstrated in Fig 4C, plasmids encoding YFP-tagged BoNT/A LC, WT SNAP-25, or SNAP-25 (1–197) were transiently transfected to HEK293 cells at equal amounts (0.5 μg of plasmid DNA), and the cell lysates were collected on days 1, 2, 3, 5, 10, and 20. PC12 cells were transiently transfected with plasmids using Lipofectamine2000 (Thermo Fisher, 11668019) or X-tremeGENE 9 DNA transfection reagents. For the protein stability experiments described in Fig 4D 1 μg of YFP-tagged BoNT/A LC plasmid DNA was transfected to PC12 cells, and cell lysates were collected on days 1, 2, 5,10,19, 21, and 23. For the membrane extraction experiments, Mem-PER Membrane Protein Extraction Kit (ThermoFisher, 89842) was used according to the manufacturer’s protocol, using HEK293 cells (Fig 4A), which were co-transfected with plasmids encoding WT SNAP-25 and SNAP-25 (1–197), or PC12 cells (Fig 4B), which were transfected with BoNT/A LC plasmid. For the thermal stability experiments of SNARE proteins presented in S2 Fig, total lysates, soluble membrane fractions, and cytosolic fractions obtained after membrane extraction were analyzed by western blotting under both boiled (5 minutes at 95°C) and non-boiled conditions.

### BoNT/A holotoxin intoxication experiments

BoNT/A1 holotoxin was purchased from MetaBiologics (Madison, WI, USA), and 10 nM BoNTA holotoxin was utilized in PC12, SH-SY5Y, and HEK293 culture experiments with a total of 72 hours of intoxication time. Mouse and human ESC-derived motor neurons were intoxicated using 500 pM BoNT/A1 holotoxin, except in Fig 2E, which utilized increasing concentrations (with 0.5 nM to 20 nM) of the toxin, and tested for a four-hour incubation period at 37°C, as these cells are more sensitive to the toxin [114,115]. Toosendanin (1 µM) and Bafilomycin A1 (10 nM), known BoNT/A inhibitors, were added to the cultures 30 minutes before the addition of BoNT/A holotoxin as positive controls [115,116].

### Immunoblotting and quantification

For immunoblotting analyses, cells were lysed in NP-40 lysis buffer (1% NP-40, 150 mM NaCl, 50 mM Tris pH 8.0, dH2O), including phosphatase and protease inhibitors. Samples were centrifuged at 13400 rcf for 20 minutes at +4°C, and the total protein content was determined using Bradford or BCA assays. Samples were boiled at 95°C for 5 minutes and then run on 12% SDS-PAGE gels and transferred onto PVDF membranes. Membranes were first blocked with 5% skimmed milk or BSA in TBS-T solutions and then blotted with primary antibody overnight at 4°C. Antibodies utilized in immunoblotting analyses included total SNAP-25 (BioLegend, 836304, RRID: AB_2566521) Phospho-Ser187 SNAP-25 (PhosphoSolutions, p1558-187, RRID: AB_2492239 or Abgent, AW5468), Phospho-Thr138 (Abgent, AP15001a), Anti-syntaxin-1A (Sigma-Aldrich, S0664, RRID: AB_477483 or BioLegend, 827001, RRID: AB_2564907), Sortilin (BD Biosciences, 612101, RRID: AB_399472), GAPDH (Santa Cruz Biotechnology, sc-365062, RRID: AB_10847862), β-actin HRP (Santa Cruz Biotechnology, sc-47778 HRP, RRID: AB_2714189), GFP (Thermo Fisher, A-11122, RRID: AB_221569 or Santa Cruz Biotechnology, sc-9996, RRID: AB_627695), HA-HRP(Roche, 12013819001, RRID: AB_390917), and Anti-HA (Sigma-Aldrich, H6908, RRID: AB_260070). β-actin and GAPDH were used as loading controls. Western blot imaging and band quantification were performed using SynGene Software, and the results are presented as mean ± SD. The quantification values of control samples were calculated, and the average control protein levels were set as 100% for normalization. Subsequently, quantification values for other experimental conditions were calculated as a ratio of their respective control values. Data was normalized to loading controls where appropriate. The extent of % pSNAP-25 Ser187 phosphorylation was normalized to the corresponding total SNAP-25 levels.

### Immunoprecipitation

For HA-Immunoprecipitations shown in Fig 5A, PC12 cells were transfected with 2 µg/well HA-tagged SNAP-25 (1–197) plasmid for 18 hours. The indicated samples were then incubated with 1µM PMA for 1.5 hours. Cells were lysed, processed, and incubated with Protein A magnetic beads (Biorad, 161–4013) and rabbit polyclonal Anti-HA antibody overnight. Normal rabbit IgG antibodies (Santa Cruz Biotechnology, sc-2027, RRID: AB_737197) served as an immunoprecipitation control. Following the incubations, the beads were washed with PBS-T 3 times, followed by the addition of 4X Laemmli sample buffer. The samples were heated at 70°C for 10 min; the beads were magnetized, and eluted proteins were analyzed by immunoblotting.

For SNAP-25 immunoprecipitations shown in Fig 5B, PC12 cells were transfected with 2 µg/well BoNT/A LC plasmid for 18h, followed by 1µM PMA treatment to the indicated samples for 1.5 hours. Cell lysates were processed and incubated with Protein G magnetic beads (BioRad, 161–4023) and mouse monoclonal anti-SNAP-25 antibody overnight. Normal mouse IgGs (Santa Cruz Biotechnology, sc-2025, RRID: AB_737182) were utilized as an immunoprecipitation control. Immunoprecipitated samples were run on 12% SDS-PAGE gels for 6 hours to separate the SNAP-25 signal from the IgG light chain band.

### Neurotransmitter release detection with mass spectrometry-based analyses

PC12 cells were transiently transfected with 1 µg YFP-tagged BoNT/A LC under conditions to achieve a low level of SNAP-25 cleavage and treated with and without 1 µM PMA for 30 minutes. To stimulate neurotransmitter release, the cells were treated with a high K^+^ solution (95 mM NaCl, 50 mM KCl, 1.2 mM KH_2_PO_4_, 2.5 mM CaCl_2_, 1.2 mM MgSO_4_, 11 mM glucose, 15 mM HEPES-Tris, pH 7.4) as demonstrated in the literature [117], and the supernatant was collected for MS analysis. Cells in each condition were lysed in NP-40 lysis buffer (1% NP-40, 150 mM NaCl, 50 mM Tris, pH 8.0, dH2O), including phosphatase and protease inhibitors for further immunoblotting analyses. Experiments were repeated, independently, three times. For the mass spectrometry analyses, which were conducted by Duzen Laboratory Group (Ankara, Turkiye), samples were prepared using solid phase extraction with Alumina, followed by elution with acetonitrile solution containing 2% formic acid (95:5; v:v). A deuterium-labeled internal standard (dopamine-D4) was incorporated for quantification. A volume of 20 µL of each sample was injected into a Waters TQ-S Micro triple quadruple LC-MS/MS system, equipped with an ACE 5 C18 column (150 × 4.6 mm). 2% methanol solution containing 0.1% formic acid was used as the mobile phase. The method parameters were set as follows: capillary voltage, 0.50 kV; drying gas flow rate, 900L/h; and drying gas temperature, 600°C. Analyte detection was performed in positive ion mode using Multiple Reaction Monitoring (MRM) mode, and the mass-to-charge ratio (m/z) for dopamine was determined as 154 > 119 ([M + H]).

### Molecular modeling

Molecular dynamics simulations were performed with the CUDA accelerated version of pmemd in Amber20 [118,119] for four different complexes. Preparation of the systems and analyses were performed with the AmberTools21 package. The starting structure for all models was the crystal structure of the SNARE complex with PDB-ID 1SFC [120]. The structure contains four SNARE complexes, and the first one was selected for all the models presented in this work. It contains VAMP2 (25–93), syntaxin-1A (188–259), and two fragments of SNAP-25, i.e., N-ter (7–83) and C-ter (131–204). The structure was used as is for the wild-type complex, which we further refer to as SNAP-25. A cleaved version of the complex was prepared by deleting residues 198–204 in the C-ter helix of SNAP-25 and is further referred to as SNAP-25 (1–197) for consistency with the experimental part of the present article. SNAP-25 and SNAP-25 (1–197) were then phosphorylated at Ser187, and the resulting models are respectively named pSNAP-25 (Ser187) and pSNAP-25 (1–197) (Ser187). All models were solvated in a truncated octahedron-shaped box of TIP4Pew water molecules [121] with a buffer region set to 10 Å. Standard amino acids were represented with the ff14SB forcefield [122], and phosphoserines were modeled with parameters adapted from the work by Homeyer et al. [123]. Each simulation box was neutralized and ionized with sodium and chloride ions to reach an approximate salt concentration of 0.15 M. Molecular dynamics simulations were performed with a time step of 2 fs with periodic boundary conditions and particle mesh Ewald to treat long-range interactions with a real space cutoff of 12.0 Å. The SHAKE algorithm was used to constrain bonds involving hydrogen atoms. A gradual heat-up procedure was used to bring the systems to a target temperature of 300 K, starting in the NVT ensemble at a low temperature and switching to NPT at 150 K and higher. Production runs were performed for 750 ns in the NPT ensemble with the Anderson thermostat (collision frequency of 2.0 ps^-1^) and the Berendsen barostat with default settings. Each simulation was replicated once, leading to a total of 1.5 μs for each complex. The backbone RMSD time series of the simulations are reported as Supplementary Information (S1 Fig).

Analysis and postprocessing of the simulations were performed on the last 300 ns of each simulation to ensure that the results are representative of an equilibrium state of the systems under investigation. Binding free energies between fragments were calculated via the MMGBSA protocol, with an in-house code allowing decomposing the energy into individual components. Only residues 54–88 of VAMP2, 224–258 of syntaxin-1A, and 51–82 and 173–203 (or 173–197 when cleaved) of SNAP-25 were included in order to focus the analysis on the local changes around the cleavage and phosphorylation sites. Analysis of selected distances was performed with cpptraj of AmberTools21. Electrostatic potential was calculated via the Delphi web server [124,125].

The helical strength of a short peptide (i.e., ACE-LYS-ALA-ASP-SER-ASN-LYS-THR-ARG-NME) was investigated via umbrella sampling to mimic the local environment of Ser187 in SNAP-25. Two peptides were studied, with canonical and phosphorylated serine. After preparation, solvation, and equilibration of the systems in the same conditions as for the large complexes described above, the distance between the α-carbon atoms of the N-ter lysine and the C-ter arginine was stretched in 17 consecutive windows from 10.0 to 18.0 Å with a step of 0.5 Å and a force constant of 16.0 kcal mol^-1^ Å^-2^ (i.e., 8.0 kcal mol^-1^ Å^-2^ in Amber’s input). The potential of mean force was then obtained via the WHAM program [126]. For each peptide, the umbrella sampling simulation was performed 50 independent times and the profiles were averaged for final analysis.

## Statistical analyses

Statistical significance was calculated using GraphPad Prism software for all statistical analyses, and the data represent mean ± SD from at least 3 biological replicates within the figures. Unpaired two-tailed Student’s t-test was utilized in the analyses of Figs 1A, 1C, 3A, 4A, 4B, 5A, and 5C, while two-way ANOVA followed by post hoc Tukey’s multiple comparison test was utilized for multiple groups in Figs 3C, 3D, 4C, and 4D. One-way ANOVA followed by post-hoc Dunnet’s multiple comparison tests was utilized in Figs 1B, 1D, 2B, 2D, and 5B, and one-way ANOVA followed by post hoc Tukey’s multiple comparison test was utilized in Figs 3B and 5D. Two-way ANOVA followed by post hoc Sidak multiple comparison test was utilized in Fig 2A. Pearson correlation with regression analyses is presented in Fig 2E. Data represent mean ± S.D. Values are significant at 99.9%, 99%, and 95% confidence levels, respectively. *: P ≤ 0.05; **: P ≤ 0.01; ***: P ≤ 0.001.

## Supporting information

S1 Fig RMSD time series of four sets of simulations of the SNARE complex. The labeling of the systems is consistent with that used in the main text. RMSDs are calculated with respect to the first frame of production based on backbone atoms only (C, CA, and N). Each simulation was performed twice. RMSD is reported for the full complexes and the region of interest discussed in the main text: i.e., residues 54–88 of VAMP2, 224–258 of syntaxin-1, and 51–82 and 173–203 (or 173–197 when cleaved) of SNAP-25.
(TIF)

S2 Fig SNARE complexes formed in the presence of PMA treatment are not heat-stable. (A) Membrane extractions were performed in the conditions described in Fig 4B. PC12 cells were transfected with BoNT/A LC in conditions to achieve a low-level SNAP-25 cleavage and treated with and without 1 µM PMA for 1.5 hours. (B) The total lysate, as well as the soluble membrane and the cytosolic fractions, were subjected to western blotting, with the indicated antibodies, following boiled (5 min at 95 ^0^C) and non-boiled conditions. Representative blots of 3 independent experiments are shown.
(TIF)
