# Supplementary figures and images for: Phosphorylation of SNAP-25 at Ser187 is enhanced following its cleavage by Botulinum Neurotoxin Serotype A, promoting the dominant-negative effect of the resulting fragment
Source: PLoS Pathog. 2025 Oct 14;21(10):e1013604. doi: 10.1371/journal.ppat.1013604 (PMC12548857; doi:10.1371/journal.ppat.1013604)

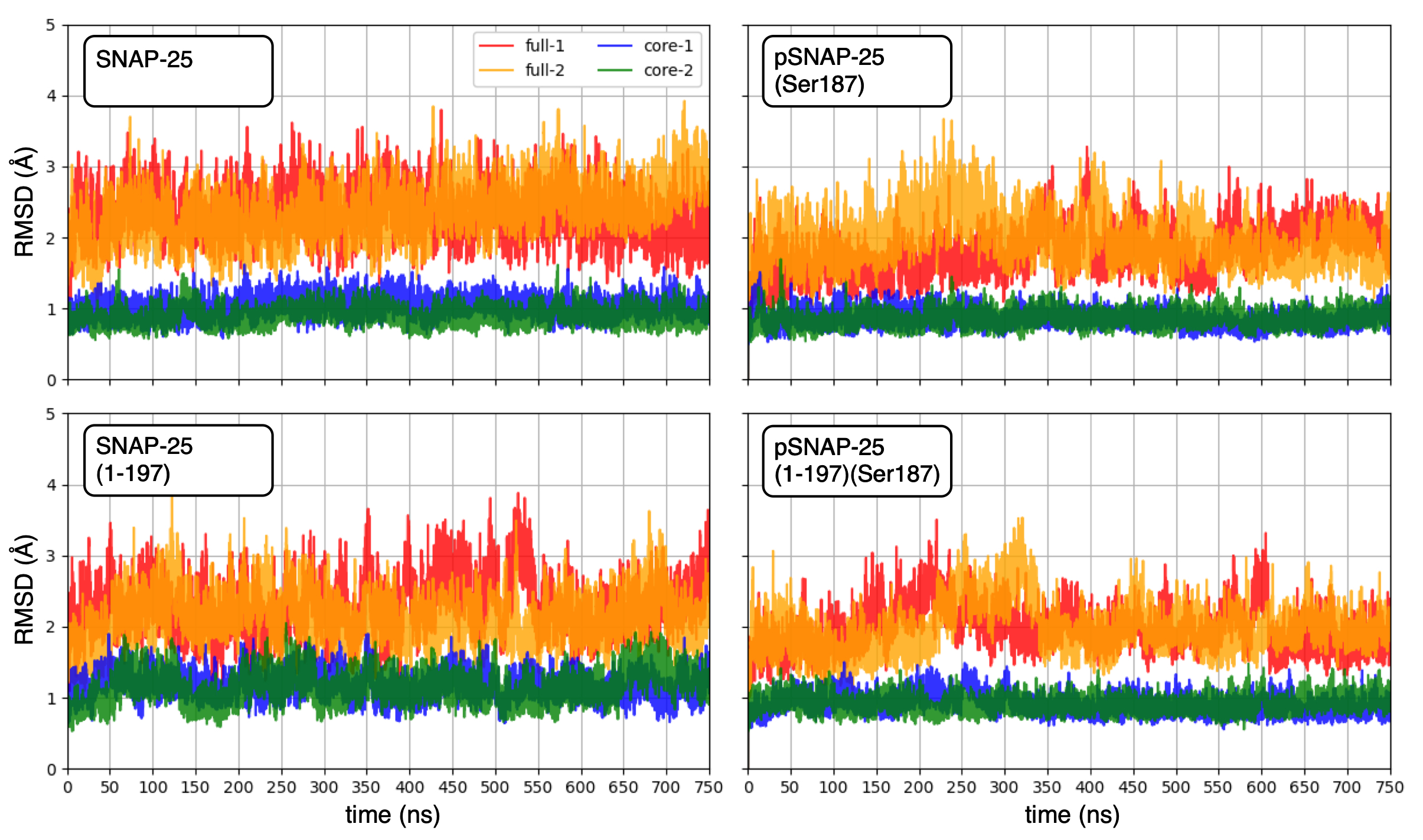

Supplement: S1 Fig — The labeling of the systems is consistent with that used in the main text. RMSDs are calculated with respect to the first frame of production based on backbone atoms only (C, CA, and N). Each simulation was performed twice. RMSD is reported for the full complexes and the region of interest discussed in the main text: i.e., residues 54–88 of VAMP2, 224–258 of syntaxin-1, and 51–82 and 173–203 (or 173–197 when cleaved) of SNAP-25. (TIF) [file ppat.1013604.s001.tif]

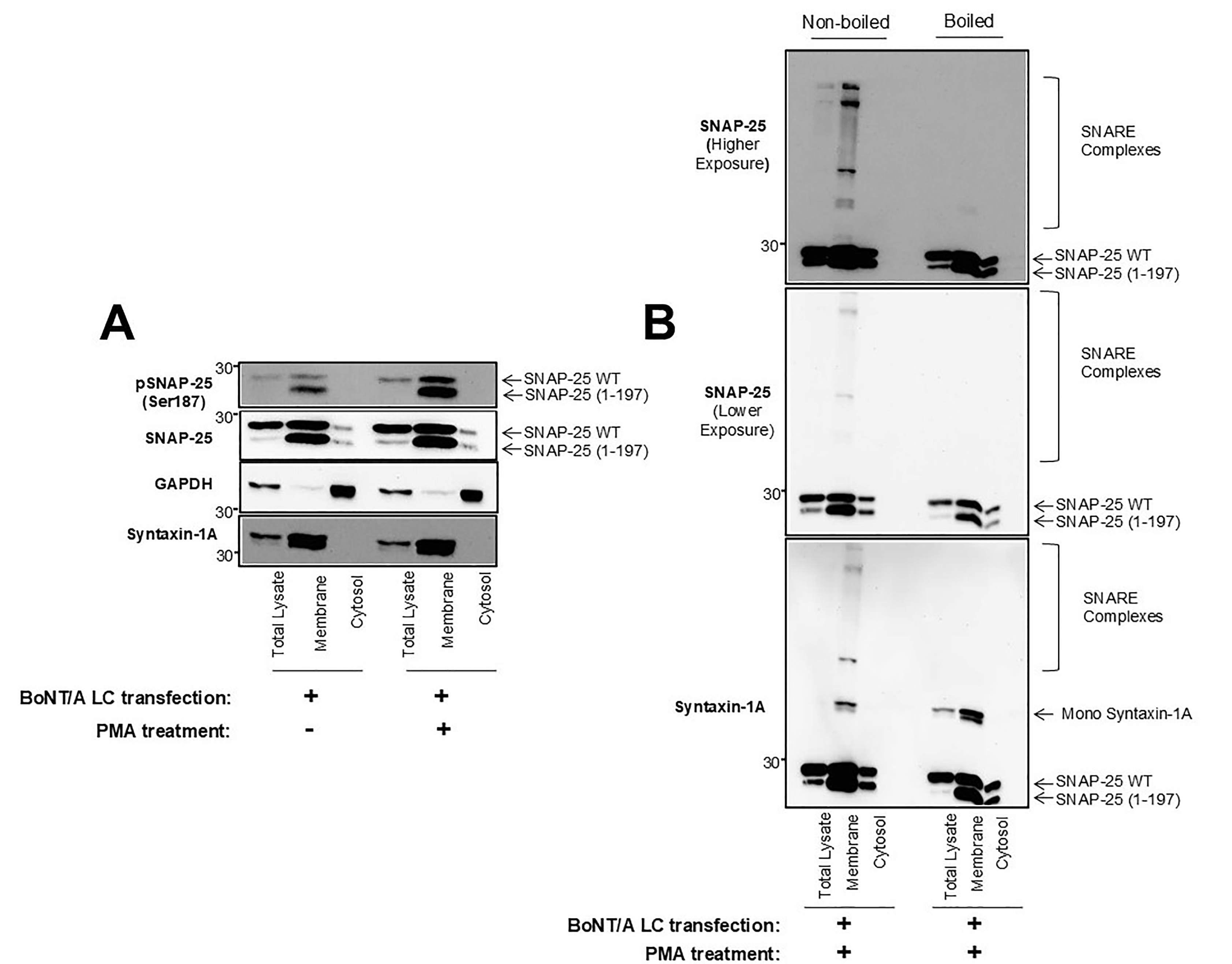

Supplement: S2 Fig — (A) Membrane extractions were performed in the conditions described in Fig 4B. PC12 cells were transfected with BoNT/A LC in conditions to achieve a low-level SNAP-25 cleavage and treated with and without 1 µM PMA for 1.5 hours. (B) The total lysate, as well as the soluble membrane and the cytosolic fractions, were subjected to western blotting, with the indicated antibodies, following boiled (5 min at 95 0C) and non-boiled conditions. Representative blots of 3 independent experiments are shown. (TIF) [file ppat.1013604.s002.tif]
